# The Role of Artificial Intelligence in Identifying Depression and Anxiety: A Comprehensive Literature Review

**DOI:** 10.7759/cureus.56472

**Published:** 2024-03-19

**Authors:** Fabeha Zafar, Laraib Fakhare Alam, Rafael R Vivas, Jada Wang, See Jia Whei, Sameer Mehmood, Amirali Sadeghzadegan, Mohit Lakkimsetti, Zahra Nazir

**Affiliations:** 1 Internal Medicine, Dow University of Health Sciences (DUHS), Karachi, PAK; 2 Internal Medicine, Ministry of Health, Kuwait, KWT; 3 Nutrition, Food and Exercise Sciences, Florida State University College of Human Sciences, Tallahassee, USA; 4 Medicine, St. George's University, Brooklyn, USA; 5 Internal Medicine, Sriwijaya University, Palembang, IDN; 6 Medicine, Jinnah Sindh Medical University, Karachi, PAK; 7 General Medicine, Marmara University School of Medicine, Istanbul, TUR; 8 Internal Medicine, Mamata Medical College, Khammam, IND; 9 Internal Medicine, Combined Military Hospital, Quetta, Quetta, PAK

**Keywords:** ai chatbot, machine learning (ml), generalized anxiety disorder (gad), depression, artificial intelligence in medicine

## Abstract

This narrative literature review undertakes a comprehensive examination of the burgeoning field, tracing the development of artificial intelligence (AI)-powered tools for depression and anxiety detection from the level of intricate algorithms to practical applications. Delivering essential mental health care services is now a significant public health priority. In recent years, AI has become a game-changer in the early identification and intervention of these pervasive mental health disorders. AI tools can potentially empower behavioral healthcare services by helping psychiatrists collect objective data on patients' progress and tasks. This study emphasizes the current understanding of AI, the different types of AI, its current use in multiple mental health disorders, advantages, disadvantages, and future potentials. As technology develops and the digitalization of the modern era increases, there will be a rise in the application of artificial intelligence in psychiatry; therefore, a comprehensive understanding will be needed. We searched PubMed, Google Scholar, and Science Direct using keywords for this. In a recent review of studies using electronic health records (EHR) with AI and machine learning techniques for diagnosing all clinical conditions, roughly 99 publications have been found. Out of these, 35 studies were identified for mental health disorders in all age groups, and among them, six studies utilized EHR data sources. By critically analyzing prominent scholarly works, we aim to illuminate the current state of this technology, exploring its successes, limitations, and future directions. In doing so, we hope to contribute to a nuanced understanding of AI's potential to revolutionize mental health diagnostics and pave the way for further research and development in this critically important domain.

## Introduction and background

Every person tends to react emotionally in a particular way, usually independent of the external stimulus. The reaction revolves around three components of emotions: subjective feelings of the individual, physiological changes, and a person's behavior to express emotions [1]. Regulation of emotions occurs through three main parts of the brain. The brainstem is responsible for an unconscious drive. The limbic system modifies the emotional response based on the environment, and the pre-fontal cortex deals with conscious emotions and feelings and controls them [2,3]. These brain areas are subject to external factors, which can cause deregulation in their functioning, eventually leading to abnormal behavioral traits. These combined changes consequently lead to a variety of mood disorders, including anxiety, depressive disorders, psychosis, and other personality disorders [4]. 

The insidious dark shadows of depression and anxiety afflict millions globally, exacting a heavy toll on individual well-being and societal productivity [5]. According to the WHO Facts Sheets 2023, 5% and 4% of the global population are affected, respectively [5,6]. Moreover, the evidence suggests that over the last decade, the incidence of depression has increased worldwide [7]. The relationship between depression and anxiety has been controversial for an extended period. Based on recent evidence, neurobiological and genetic similarities have been seen between the two. Approximately 85% of patients with depression are shown to exhibit significant symptoms of anxiety. Similarly, in about 90% of patients with anxiety disorders, comorbid depression is seen [8,9]. 

Artificial intelligence (AI) has recently emerged as a potential game-changer in the early identification and intervention of these pervasive mental health disorders [10-16]. While the application of this technology is growing, some of the more concrete applications of AI in mental health for identifying and managing depression and anxiety include early detection and risk assessment, improved diagnosis and assessment, personalized treatment and intervention, and research and development [10-20].

Various tests can be performed using former techniques and machine learning (ML) algorithms to detect emotional imbalances under different scenarios. Interestingly, this has led to the development of emotionally intelligent machines based on various Al-based approaches to detect emotions in human beings, depression being one of them. 

Text-based emotion recognition uses machine learning algorithms like Naive-Bayes and support vector machines (SVM). Principal component analysis (PCA) is a machine-learning technique that detects emotions through facial expressions, speech, and gestures. An emotion-detecting system is used in video gaming and customer reviews to detect fear/excitement in the player and the customer's emotions, respectively [21]. Certain companies also use emotional analytics during the recruitment process for candidate selection. Haar-cascade algorithms, K-nearest neighbors (KNN) classification technique, and optical character reader (OCR) are a few techniques that use AI and artificial neural networks to detect depression through facial expression. Partial least square algorithm detects emotion through vocal stimuli. ML techniques, for example, term frequency-inverse document frequency (TF-IDF), long term short term (LTST) - radial neural networks (RNN), logistic regression, and linear support vector, are used to detect depression through tweets. Sentiment analysis with the use of the natural language processing (NLP) technique is used in emotion recognition in tweets [21].

Other applications of AI in screening, diagnosis, and treatment

Chatbots and Virtual Assistants

Chatbots and virtual assistants can conduct initial patient screenings and assess symptom severity through personalized conversational interactions [17].

Wearable and Mobile Sensors

Monitoring sleep patterns, activity levels, heart rate, and voice intonation through wearable devices can provide objective data for identifying early symptoms [11,13,19].

Neuroimaging Analysis

AI can analyze brain scans to identify potential biomarkers for depression and anxiety.

Tailored Therapy Recommendations

AI algorithms can analyze a patient's data and suggest personalized treatment plans.

Remote Diagnosis, Monitoring, and Support

AI-powered chatbots and interactive virtual agents can assist depression detection and provide ongoing support and monitoring, improving treatment adherence and engagement [21].

Digital Therapeutic Interventions

AI-powered apps and programs can provide cognitive behavioral therapy (CBT), mindfulness exercises, and other evidence-based interventions for self-management of depression and anxiety; for instance, Youper is a mobile app used in the treatment of depression and anxiety [22].

Predicting Treatment Outcomes

AI can help interpret patients that might respond to specific interventions, furthering personalized medical approaches.

In addition to AI's benefits to mental health, its application has a few challenges. These include a lack of accountability and a lack of standard ethical and legal framework [23]. This narrative literature review undertakes a comprehensive examination of the burgeoning field, tracing the development of AI-powered tools for depression, anxiety diagnosis, and treatment from the level of intricate algorithms to practical applications. By critically analyzing prominent scholarly works, we aim to illuminate the current state of this technology, exploring its successes, limitations, and future directions. In doing so, we hope to contribute to a nuanced understanding of AI's potential to revolutionize mental health diagnostics and pave the way for further research and development in this critically important domain. 

## Review

Artificial intelligence has emerged as one of the most critical developments in mental health. To properly analyze its application in the screening, diagnosis, and treatment of depression and anxiety, it is essential first to understand the current tools used for this purpose. Accurate diagnosis is crucial when addressing anxiety and depression, this introduction offers an overview of the current diagnostic tools used in clinical practice to identify and assess these disorders, providing insights into medical professionals' methods for diagnosis. Screening individuals for depression can assist in identifying those who require intervention, leading to enhancements in their well-being and overall clinical condition. Concise screening questionnaire tools can be administered with minimal personnel [24]. Conducting screening activities requires training and proficiency, given that identifying depression symptoms can be challenging due to concurrent medical conditions such as pain, cognitive impairment, anxiety, and disability [25].

Major depressive disorder (MDD) is characterized by a range of symptoms that are not dependent on age [26]. To be diagnosed with MDD, an individual must exhibit a persistently sad mood and reduced interest or enjoyment in activities. Additionally, they must experience at least four of the following symptoms for a minimum of two weeks [27]: changes to appetite or body weight, sleep disturbances, restlessness or a sense of slowness, overwhelming fatigue or a persistent lack of energy, difficulty concentrating or making decisions, feelings of unworthiness or inappropriate guilt, contemplation of death or suicidal ideation. 

In depression screening, two widely utilized tools, the Patient Health Questionnaire-2 (PHQ-2) and the extended Patient Health Questionnaire-9 (PHQ-9), play vital roles in assessing and evaluating depressive symptoms [28]. The scale employs a four-point response system with the following options: 0 (no days), 1 (some days), 2 (more than half of the days), and 3 (almost every day) [29]. To enhance the reliability and validity of depression screening, the PHQ-9 can be used in conjunction with the PHQ-2, providing a comprehensive evaluation [30]. The PHQ-9 extends beyond the PHQ-2 by encompassing all nine symptoms of major depression. An efficient approach involves administering the PHQ-2 and, for those with a positive initial screen, assessing the remaining seven symptoms. Higher PHQ-9 scores indicate greater depression severity, although the score ranges from 0 to 27 [28].

Generalized anxiety disorder (GAD) can be diagnosed using the screening tool form of GAD-7 [24,31]. The Diagnostic and Statistical Manual of Mental Disorders (DSM-5) outlines the diagnostic criteria for GAD. To be diagnosed with GAD, an individual must experience inappropriate anxiety excessive in intensity for at least six months with accompanying symptoms of restlessness or feeling on edge, easily tired, difficulty concentrating, irritability, muscle tension, and sleep disturbances.

The GAD Scale employs a Likert-type rating system featuring four response choices, ranging from 0 (not at all), 1 (several days), 2 (more than half the days), and 3 (almost every day) [32]. This scale assesses anxiety symptoms experienced over the past two weeks, aligning with DSM-IV criteria. The GAD-7 yields scores from 0 to 21 [32]. The GAD-7 provides a foundation for diagnosing patients with GAD, but keep in mind that there are other types of anxiety disorders, such as phobia, social anxiety, panic disorder, and agoraphobia [31]. However, a formal diagnosis can only be made if another underlying medical condition does not explain the symptoms, and healthcare providers must consider the multifactorial causes of health conditions. 

Medical diagnostics is the name given to the practice of analyzing patients' symptoms, medical histories, and test results to evaluate potential medical conditions or diseases. As a rule, diagnostic medicine aims to establish what causes an individual health issue to plan the proper treatment. Medical diagnostics often entail diverse diagnostic procedures, including blood test imaging techniques such as X-rays, CT scans, MRI scans, biopsies, and many more. 

Following the results of these reports, healthcare providers determine the best course of treatment for their patients. Aside from diagnosing health problems, medical diagnostics can also be employed to track the improvement of a condition, evaluate the effectiveness of a therapy, and identify early signs of problems before they become serious [33]. There is vast scope for AI to transform mental health; it is built on the foundations of individualized patient care and availability [10,34]. AI-powered mental health applications aid in the early identification of mental health disorders, offering personalized treatment and support [10,35,36]. Utilizing these applications does not call for in-person appointments and long waiting periods by offering continuous and seamless support around the clock, thereby providing practical progress in the treatment's effectiveness [10]. AI allows machines and computers to make decisions by learning from data. AI has played a role in revolutionizing mental health support by providing accessible and personalized care to individuals. 

Using Electronic Health Record and novel machine learning approach with AI for diagnosis and treatment of depression and anxiety

Depression and anxiety are highly prevalent mental health disorders, but sadly, many cases are left untreated due to poor identification of risk factors and warning signs. With the recent developments and advancements in research and healthcare, various new methods have evolved to diagnose and treat the above two. With the use of AI, one of the methods in early identification of depression and anxiety is using primary care Electronic Health Record (EHR) [37].

Administrative healthcare data (AHD) and EHR are used in geriatric mental health research. However, alternative analytic approaches such as ML with AI come into play due to the data's increasing amount and complexity [38]. ML utilizes already established algorithms to perform tasks without specific programmed instructions while continuously learning from the tasks performed.

Analytic strategies employed with EHR and AHD studies involve regression models such as logistic regression, linear regression, or time-to-event models like Cox-proportional hazard models. There is also a growing interest in the potential applications of ML and AI in data analysis of EHR and AHD in mental health studies [10,39]. AI and ML may provide benefits compared to standard biostatic regression analysis, especially when there is high complexity to the underlying data, which has become more familiar with AHD and EHR data as a great range of information is included in these data sources [40].

The application of AI and ML to EHR and AHD analysis is evolving with time, including developing recommendations for using the former two with these datasets and including their studies in biomedical research [41,42]. A recent review of studies using EHR with AI and ML techniques for diagnosis or classification across all clinical conditions has identified about 99 unique publications [43]. Out of these, 28 studies were identified for mental health disorders in all age groups, and among them, six studies utilized EHR data sources [10]. EHR and AHD are rich resources that gather information related to patients' healthcare records and allow us to facilitate this knowledge for research, including mental health. However, obstacles and challenges are associated with using the above two data due to the large sample size, incompleteness, the volume of longitudinal data, and inconsistency [43].

ML and AI can also be used to analyze unstructured data, for example, free-text clinical notes, which are increasingly available in EHR. Incorporation of clinician-generated data from unstructured data sources substantially improves predictive performance as compared to analyses that involve only structured data, for example, laboratory values. This highlights the potential future for research and clinical applications related to EHR and AHD in mental health diseases [44]. ML is used to develop models that assess the probability of individuals developing mental health conditions by analyzing various factors such as genetics, lifestyle, and environmental data [45,46]. Prior research has relied on ML using classification techniques to detect mental health problems [47]. The most commonly used ML techniques in mental health disorder detection include SVM, logistic regression, random forests, decision trees, and artificial neural networks [47]. SVM is a type of supervised learning that falls under standard ML techniques and deals with classifications and regressions [48]. SVM relies on data mining and solves linear and nonlinear classification problems, meaning this technique can handle structured and semi-structured data. Studies done in 2015 [49] and 2017 [50] indicated that SVM has an accuracy of approximately 70%, and another study in 2017 for depression detection showed SVM accuracy of 79% [51]. AI and ML may also affect mental health disease onset, prediction, or progression. Precision medicine, which predicts treatment response and personalizes therapeutic interventions for individuals, is another potential application of the former [52]. Using patients' responses to previous treatments and genetic profiles, ML can recommend personalized therapies and medications deemed more likely effective based on the provided data [53]. Utilizing AI improves treatment outcomes and reduces the trial-and-error approach in mental health care. 

How machine learning and natural language processing contribute to AI in mental health

ML and NLP have an interlocking relationship in artificial intelligence, especially regarding mental health. NLP is a subset of ML that allows machines to understand and generate human language [54]. NLP's linguistic abilities include extracting information from textual data. ML complements NLP's abilities by learning and making informed decisions from data [54]. The collaboration between NLP and ML allows medical professionals to navigate vast data accurately. ML algorithm techniques can help with the early detection of mental health disorders via analyzing data resources such as health records and changes in speech and text patterns [45]. For example, changes in linguistic patterns or the sentiment of texts can be indicative of signs of mental health disorders [46]. NLP enables AI systems to analyze, process, and understand texts and speech similarly to humans [55]. NLP allows AI systems to respond in human language [56]. NLP is adequate at analyzing the emotional tone of texts. For example, chatbots can analyze user inputs to detect signs of distress, anxiety, or depression [57]. From there, chatbots can offer appropriate support or intervention [57]. NLP can be applied directly to individual patient data to predict suicide risk and identify disorders and comorbidities for example Boamente Program uses user's text data via smart phone application to predict suicide ideation [55]. NLP can also be used in health records to automate chart reviews, classify patients, and predict patient-specific outcomes or overall population trends [55]. NLP-powered chatbots are AI-driven systems that engage in individual conversations and can offer human-like, empathetic responses and guidance. The most cost-effective natural language processing is smartphone data, which is easily accessible and contains valuable personal data for analyzing patterns linked to behavioral changes [12,58]. NLP and ML work together in AI-directed mental health care [59]. NLP techniques extract information and enable ML algorithms to analyze data for trends, risk factors, and potential issues [60].

Categories of artificial intelligence-based diagnostic and therapeutic tools for depression and anxiety

**Figure 1:**
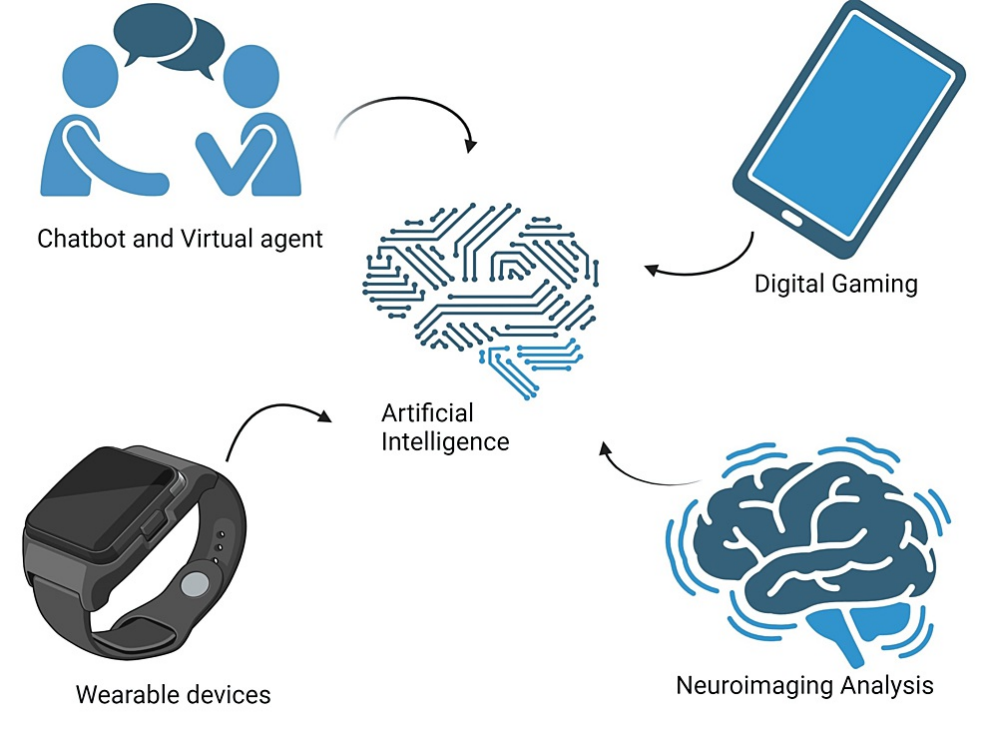
Artificial Intelligence and its use in various categories Created with BioRender.com

Following are a few examples of application of AI in the diagnosis and treatment of depression and anxiety. It's implication has been mentioned in Figure 1 as well.

Chatbot and virtual agents

In the pandemic coronavirus disease 2019 (COVID-19) era, chatbots have been created to assist and enhance mental health care support. AI chatbots have had an increased demand throughout the years. One of their essential usages is their assistance in therapy for mental disorders, with depression being the most common [61]. They play a pivotal role in enhancing the quality of patient responses and can engage with individuals who prefer alternatives to in-person therapy and spare time for medical professionals [62]. AI chatbots have the potential to simulate psychotherapist interactions, assess individual depression levels, and suggest self-help strategies [63]. Additionally, AI chatbots can efficiently handle vast databases of diseases, symptoms, and treatment options [64]. Chatbots assist in diagnosing by asking questions about mood and stress [65-68]. A few examples of chatbots are as follows:

Woebot

One of the chatbots invented is Woebot, which was specifically made to provide cognitive behavioral therapy (CBT) tools through social media or mobile applications. CBT tool is one of the ways to manage and identify challenging mental health issues. A randomized controlled trial was conducted with 70 subjects, and it was concluded that there was a significant decrease in the Woebot group users [35,66].

Tess

Tess uses text messaging to coach individuals to overcome their mental distress and to provide therapeutic conversations and ways to cope with their mental health issues [66,68].

Replika

Replika is a smartphone app that allows users to communicate with an avatar for self-reflection. It allows users to be vulnerable, to open up without constant fear of judgment, and to improve their personality in a better direction [68,69].

AI-Enabled Companion Bots

There are also companion bots like Paro and eBear that provide animal therapy to assist with mental health issues such as depression. They teach ways to cope and overcome mental health challenges [65,66,68].

Wearable devices

Electronic devices, such as wearable devices with various sensors and technologies, can be worn on the body. The primary goal of these wearables is to provide users with real-time information about their health, activities, or environment. Integrating wearable devices with artificial intelligence has emerged as one of the most significant advancements in AI for screening, diagnosing, and managing depression and anxiety [70-72]. There is a scarcity of mental health practitioners worldwide. Statistics have shown that there are only around nine psychiatrists per 100,000 in high-income countries. In contrast, in low-income countries, the ratio is as low as 0.1 psychiatrists per one million people [73,74]. Hence, developing automated techniques is vital to address this shortage. Numerous wearable devices are available, including but not limited to smartwatches, smart bands, bright shirts, and smart glasses. The most common device used by most research studies has been an intelligent band [70,75,76]. This can be explained by the fact that they are less distracting and easy to use, which makes them more user-friendly [77]. These wearable devices continuously record users’ parameters such as step count (physical activity), heart rate, sleep data, temperature, and blood oxygen. These parameters are significant because studies have shown that depression is associated with a decrease in physical activity, whereas treatment with antidepressants has been shown to increase activity levels significantly [78-80]. Additionally, increased activity is also related to lower depression [81,82]. 

**Figure 2:**
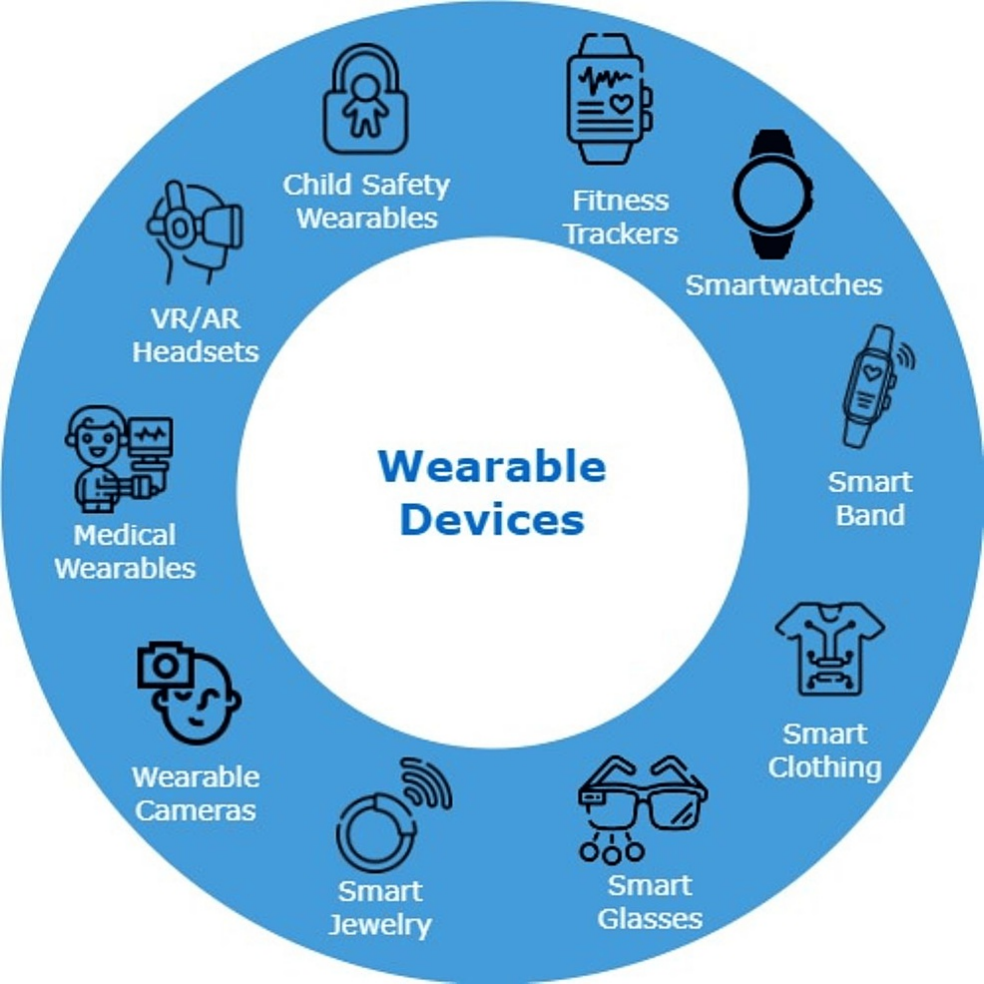
The available wearable devices AR: augmented reality, VR: virtual reality Figure created using flaticon.com

The association between sleep patterns and depression and anxiety is also well-established [75,83,84]. The relationship of heart rate and heart rate variability with depression and anxiety is also well known [85-87]. Furthermore, it has also been reported that higher body temperature is associated with depression [88,89]. Figure 2 highlights all available wearable devices. 

Most studies have shown that wearable AI is often used for diagnosis and screening of anxiety and depression [13,70,75]. These devices can continuously record data and track real-time changes. This data can be integrated with AI, used for screening and diagnosis of depression and anxiety, and utilized for its management. This can help in early diagnosis of depression and anxiety-related symptoms, which in turn can help to prevent depression in individuals [13]. Pre-screening evaluation can also be done via these devices, and individuals can be notified of needing a mental health checkup. 

**Figure 3:**
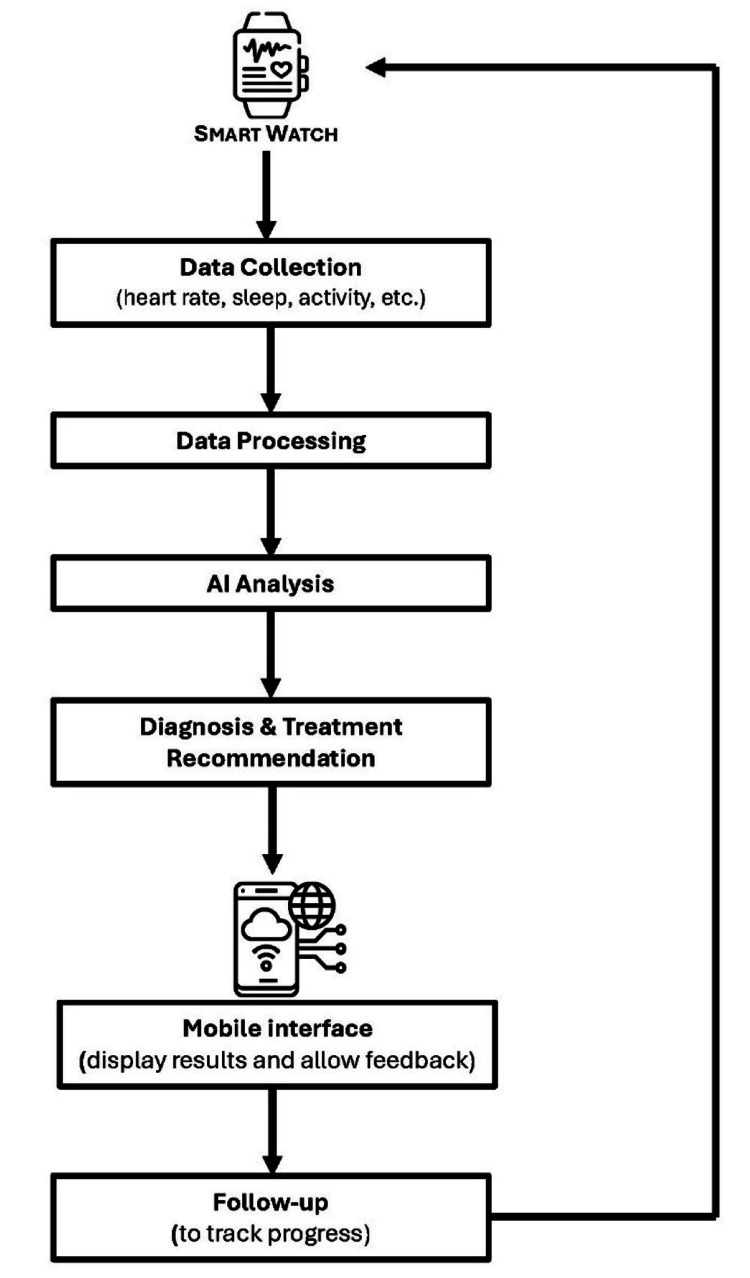
Introducing artificial intelligence (AI) in wearable devices Source Reference no: [71,73]

Treatment via the help of wearable devices still needs to be investigated, although some interventions for treatment purposes, including mindfulness and biofeedback therapy, have been studied [76,90]. However, there is still a huge research gap in this field. Hence, further studies and interventions are required to use AI-integrated wearable technology in the treatment of depression and anxiety. Figure 3 highlights how AI is implemented in wearable devices.

AI in digital gaming interventions and smartphone applications

AI has also played a part in digital gaming interventions [66,68,91]. In the past, digital gaming was used to detect symptoms. Psychoeducation has now evolved into complete programs that improve the psychosocial and cognitive domains of various mental health issues. Many therapeutic services include cognitive behavioral therapy, behavioral modification, social motivation, attention enhancement, and biofeedback. This is appealing to users as games are now easily accessible via smartphones and can improve their user’s mental health. Other than that, several smartphone applications have been invented to use AI, such as mindLAMP and BiAffect, which help with mental health conditions such as depression and anxiety to help predict recovery through various data and to assist them in overcoming mental health challenges [68,91].

Neuroimaging analysis

The increase in artificial intelligence capabilities, especially machine learning algorithms, is a powerful tool for automation neuroimaging data analysis. Complex patterns may be investigated and resolved in the data details of the voluminous databases, looking back at long-standing research that can make human observers also disseminate publications for literature review or neurotic council. This automation saves time and eliminates human error and inter-observer variability, as is often the analysis case. Many studies have shown AI algorithms' ability to analyze neuroimaging data automatically. For instance, machine learning models have been successfully used to identify specific brain regions involved with anxiety and depression, such as the amygdala, anterior cingulate cortex, and prefrontal cortex [92]. AI algorithms hold a significant impact on the detection and effective removal of biomarkers linked to anxiety and depression. Through analyzing detailed information, these algorithms can reveal microstructural, functional, or connectivity differences in the brain that could be potential biomarkers of such diseases. For instance, abnormal connection patterns have been identified as specificity factors in anxiety disorders [93]. Such biomarkers can be detected and quantified by AI algorithms with a high level of accuracy, enabling the refinement of diagnostic criteria. AI can also identify biomarkers and differentiate between various anxiety or depression subtypes. For example, machine-learning approaches have revealed that neuroimaging of major depression and bipolar disorder can differ in species [94]. This implies that AI algorithms can deliver more reliable and personalized diagnoses, which improves treatment planning accuracy.

Personalized treatment and intervention of AI in identifying anxiety and depression

Personalization is a much-discussed approach to improving compliance and outcomes for digital mental health interventions (DMHIs). DMHI’s unique delivery channel provides new ways to improve the management of those suffering from anxiety and depression. The significance of accommodating patient’s preferences for treatment outcomes in mental healthcare has been well established [95]. Therefore, personalizing treatments and interventions to individual needs is a promising approach to improving care for mental health illnesses and beyond [96-98].

The main objective of personalized treatment and intervention applied in mental health is to improve precision in disease diagnosis, treatment choices, responses, and prognosis. Diverse approaches and techniques, namely genomics, epi-genomics, neural circuits, and AI, are related to precision psychiatry. Using computational and biological tools to find potential biomarkers, patients with the same endo-phenotype will likely receive biomarker-based treatment and management, thus leading to a better prognosis [99]. Digital therapeutics (DTx) involves qualified software programs to manage, prevent, and treat medical conditions and is an emerging type of medical therapy. The products and devices that fall under this, such as wearable devices, smartphones, e-therapy, and chatbots, have been developed for a wide range of medical conditions such as diabetes, oncology treatment and management, and neuropsychiatric disorders, including anxiety and depression. One of the main advantages of DTx with AI is that it can be more flexible compared to other treatment methods to address patients’ individual needs. DTx is developed for specific medical conditions, based on science and evidence-based clinical medicine, and approved by the FDA [100].

DTx can be used either as monotherapy or in combination with other forms of therapy, like medications, to improve outcomes [101]. With technological advancements, research has explored the use of AI in managing mental disorders with a personalized approach and treatment plan. For instance, technology-based behavioral sensing is promising in measuring subjective functioning, guiding management and treatment, and making inferences concerning symptoms. An example is the self-help tool, downloaded through mobile apps [102].

Research supports the effectiveness of internet-based interventions in the self-management of depression, along with symptom-specific interventions. For example, Deprexi’s program is relevant to symptomatic improvement in mild to moderate cases [103]. Such findings support the need for increased specificity in designing automated self-help programs [104]. Therefore, the treatment plan can be tailored as per the person’s symptoms, preferences, and needs when considering the AI approach rather than conventional medicine. However, it is too early to draw firm conclusions regarding the effectiveness of these treatments based on the evidence of their efficacy [105].

Behavioural and lifestyle changes for outpatient compliance (wearable devices, digital therapies and follow-up of mood monitoring)

In the past decade or two we have seen an implication of AI in clinical and research medicine, which has led to a shift from the traditional face-face consultation to technology driven interventions or e-therapies. Recent reviews have shown e-therapies to be growing in popularity among people as well as being effective [106]. A potential hurdle seen in evaluating these technologies is user compliance [107]. Not much is known about the degree to which user’s engagement aligns with the usage patterns for which these technologies are designed. Also, little is known regarding the influence of technology adherence on outcomes. In medicine literature, compliance- “The extent to which a person’s behaviour, that is- Taking medications, following a diet and/or executing lifestyle changes, corresponds with the agreed recommendations by a health care provider” persistence, and the act of compliance for the advised duration of time, are widely studied [108,109]. These behavioural variables are seen to significantly impact medical and psychotherapy outcomes [110]. In e-therapies the equivalent of failing to persist with therapy is treatment drop out, that is- when a user prematurely discontinues the use of intervention/technology. Appreciation of persistence and adherence is significant in evaluating e-therapy, including an understanding of compliance to the program, such as completion of modules and/ or e/online activities. As the field of e-therapy has been evolving, so has the interest in potentially modifiable factors that may affect compliance [110].

Program usability testing is an important factor. According to recent studies, the increased use of computer relational skills, such as use of social dialogue and empathy in computer programs, eventually leads to an increased usage of these devices and programs [111]. Several authors have started to explore the effect of trial factors and reminders on cessation of usage and thus compliance. Clarke and Colleagues comparison of overcoming depression concluded that people were more likely to demonstrate compliance if they received reminders [112]. For outpatient compliance, engagement in e-therapy and smart devices requires more cognitive, physical and time investment by the person as compared to the relative ease of taking medication daily. This may answer as to why e-therapy users are more prone to non-adherence. Compliance can be measure through objective and subjective methods. Objectives measures include the time spent online, frequency of the program being accessed by the user, number of completed activities and the patterns of usage. While subjective measures include the completion of behaviour-based activities, usage of skills along with the time spent online.

Compliance with e-therapy was also seen to be influenced by their design, application and logistics, the same way medication dosing affects outcomes [113]. Therefore, adherence/compliance is essential in understanding how these therapies may benefit individuals who need intervention. The most common use of e-therapy is to intervene in anxiety and depression.

In a randomized controlled trial (RCT), therapy provided with Eleos Health showed superior anxiety and depression outcomes, compared to TAU. These findings indicated that complementing mental health services with an AI platform specializing in behavioural treatment was more effective in reducing key symptoms compared to standard therapy [113]. With studies and research, we can see that e-therapy and AI embedded technologies such as smartphones and wearable devices have the potential to detect and monitor anxiety and depression, however, are not advanced enough for clinical use. Until further evidence demonstrates an ideal performance of the above, they should be used along with other clinical assessments.

Advantages of utilizing AI for diagnosis and treatment of depression and anxiety

Traditional methods of identifying depression and anxiety are often reliant on self-reporting and clinician assessments. These methods suffer from inherent limitations - stigma, social desirability bias, and underdiagnoses are just a few hurdles hindering timely intervention [10,14]. As the development of larger and deeper AI networks continues development at neck-breaking speed, the potential of AI in diagnosing depression and anxiety, along with other mental health disorders, flickers on the horizon. 

AI algorithms, trained on vast datasets of behavioral and linguistic patterns, can theoretically analyze speech, text, and even facial expressions to detect the subtle telltale signs of these widespread mental health concerns [19,20]. Moreover, by identifying subtle behavioral and linguistic changes associated with depression and anxiety, AI algorithms can potentially detect and diagnose these conditions earlier and with greater accuracy, paving the way for improved outcomes. This early detection holds immense promise for preventing symptom escalation, reducing healthcare burdens, and ultimately, transforming the lives of millions struggling with these debilitating conditions. Therefore, the landscape of mental health diagnosis is poised for a transformative shift with the emergence of artificial intelligence [20].

AI algorithms can go over tremendous amounts of data to identify patterns and trends invisible to the human eye, offering clinicians a more holistic understanding of their patients' conditions. This data-driven approach can inform personalized treatment plans, tailoring interventions to individual needs and preferences [114]. Furthermore, AI can also help people with mental health issues who find it challenging to have human interactions, especially with people who are afraid of social stigma, by utilizing AI through virtual therapists, chatbots, or other channels, they can seek help privately and provide convenience to their daily lives [12]. Additionally, AI can provide valuable support in tasks like symptom monitoring and early detection of relapse, allowing clinicians to focus on building therapeutic relationships and delivering high-quality care as AI techniques will redefine mental illnesses more objectively than current practices such as the DSM-5 [10,115].

Limitations of using AI for screening and diagnosis of depression and anxiety

The implementation of AI in the sector of mental health possesses many challenges related to safety and security of data, autonomy of patients, and effectiveness of its use [66,116,117]. As AI uses different external servers for storing data, there is always a risk of security breach and leaking of personal data [75]. Additionally, accuracy of diagnosing depression and anxiety using AI tools is still questionable for example wearable AI devices are not always accurate and hence the diagnosis made using the data provided by such devices cannot be relied on [75]. This in turn leads to another major concern, that is, there is no clarity of accountability when an AI system makes an error, hence patient won’t know who to hold accountable if there is any wrong diagnosis or treatment, even if it leads to detrimental effects on patients’ health [118]. Similarly, patients’ autonomy is also important to be taken care of, but unfortunately AI tools have failed to prove their transparency [119].

The inherent complexity of mental health, characterized by overlapping symptoms and individual variability, poses a significant challenge for AI algorithms to navigate. It is a well-known fact that psychiatry is a field where doctor-patient relationship, understanding and compassion is crucial for diagnosis and treatment [118]. The drawback of using AI is that it lacks these essential skills, hence, can lead to wrong diagnosis. Likewise, when using AI wearable devices, they can only record physiological data and cannot determine its subjective nature, for instance a smart watch can detect changes in heart rate but cannot determine what has caused that. Correct interpretation of such data is essential, similarly considering the emotions of patients is also very important and a crucial part of treatment, which can currently only be done by a mental health provider and not a robot or computer. Therefore, the use of AI in psychiatry is limited to being a complementary tool which cannot completely replace the role of trained mental health practitioners for diagnosis and treatment of depression and anxiety [120].

Acceptability of AI amongst patients also limits its use, as some cultures still find it disrespectful to be treated by a machine [121]. Another major issue is limited availability of technology in resource-limited areas, and lack of training for using such technologies amongst health care professionals of such areas [122]. Therefore, such technology can only be used in developed areas and hence deprive underprivileged areas of equal share in healthcare facilities. Furthermore, another important concern is that use of AI might make patients overly dependent on technology which can in turn lead to avoidance of face-to-face interactions with health care providers. Moreover, biases embedded within training data can lead to discriminatory outcomes, particularly for the marginalized groups [123]. Most importantly, there is still a lack of legal regulations, standard guidelines, and an unanimously accepted framework for use of AI in medical field [75]. Such guidelines and laws are vitally important for widespread use and acceptability of AI in the field of mental health and to maintain standard protocol of its use.

Future of artificial intelligence in mental health

The journey from algorithms to applications for AI in identifying depression and anxiety is just beginning. The actual value of AI lies in its translation from theoretical promises to practical applications. Integrating AI tools into clinical settings holds immense promise for improving patient care. Imagine AI-powered screening tools used during routine check-ups, providing initial assessments, and flagging potential cases for further evaluation. Telehealth platforms could leverage AI to offer real-time emotional support and personalized interventions, bridging geographical and accessibility barriers. Additionally, AI could analyse vast amounts of clinical data to identify patterns and inform the development of more targeted and effective treatment plans. 

However, bridging the gap between potential and practice requires careful consideration. Responsible and ethical AI implementation in mental health requires meticulous data collection practices, transparent model development, and ongoing validation studies to address these challenges and ensure equitable access to accurate and reliable AI-powered diagnostics [115]. Ensuring the affordability and accessibility of AI-powered tools is paramount to reaching individuals who need them most. Mitigating algorithmic bias and addressing data privacy and security concerns are also essential for building trust and encouraging patient engagement with these innovative technologies [14,124]. Moreover, clinicians must have the knowledge and skills to interpret and integrate AI-generated insights into their clinical practice. By prioritizing ethical considerations, fostering robust partnerships, and developing user-friendly and accessible tools, the bridge between research and clinical practice can be successfully navigated, bringing the benefits of early detection and intervention to a broader population [124].

Integrating AI into existing healthcare systems requires seamless data sharing and collaboration between mental health professionals and tech developers [125]. The current potential of AI in mental health lies not in replacing clinicians but rather in empowering them with valuable insights and tools. Ultimately, the successful integration of AI into clinical practice requires a collaborative approach, where clinicians leverage the power of AI to augment their expertise, which improves patient outcomes and a more efficient healthcare system. Therefore, the future of mental health diagnosis and treatment lies in a collaborative approach where AI and human expertise work in tandem. By fostering collaboration and harnessing the strengths of humans and machines, we can work towards a future where mental health support is accessible.

## Conclusions

From various research studies, we conclude that AI can be used to diagnose disease, develop personalized treatment plans, and assist clinicians in decision-making. Another benefit linked with AI technology is enhancing patient care across healthcare settings rather than just automating tasks, as one would assume. However, certain drawbacks and challenges are faced with its use, such as the need for human expertise, data privacy, and bias. These should be addressed for the effective and responsible implementation of AI in healthcare. 
